# Next-generation sequencing of the complete mitochondrial genome of the Nixi chicken (Gallus gallus)

**DOI:** 10.1080/23802359.2020.1810593

**Published:** 2020-08-31

**Authors:** Jingjing Gu, Sheng Li

**Affiliations:** a College of Animal Science and Technology, Hunan Agricultural University, Changsha, China; b Hunan Key Laboratory for Genetic Improvement of Animals, Changsha, China; c Hunan Engineering Research Center of Poultry Production Safety, Changsha, China; d Maxun Biotechnology Institute, Changsha, China

**Keywords:** Nixi chicken, next-generation sequencing, mitochondrial genome

## Abstract

Nixi chicken is a type of Tibetan chickens who lives at high altitudes in China. In this study, we reported the first complete mitochondrial genome of Nixi chicken by using a next-generation sequencing method. The assembled complete mitogenome contains one control region, 2 ribosomal RNAs, 13 protein-coding genes and 22 transfer RNA genes. Our work provides a valuable source of data for the study of the evolution of the Gallus gallus mitochondrial genome and contributes to the Nixi chicken breeding improvement program.

Nixi chicken is one of the valuable Chinese chicken breeds and belongs to a type of Tibetan chickens who lives at high altitudes. Nixi chicken has a small body size and can adapt well to the local natural environment of high altitude and cold climate. In this study, we sequenced and assembled the complete mitochondrial genome of Nixi chicken using the next-generation sequencing technology for the first time. The Nixi chicken was sampled at its breeding center–Shangri-la County (27.82 N and 99.70 E), Yunnan Province, China. The Nixi chicken specimen (Voucher No. NX152146) was stored at −80 °C in the Museum of Hunan provincial key laboratory for genetic improvement of domestic animal, Changsha, China for long-term usage. The raw sequencing reads of Nixi chicken were 9.8 Gb in total and were uploaded onto NCBI Sequence Read Archive (SRA) with accession number SRR4302064. The assembled complete mitochondrial genome of Nixi chicken has been deposited in Genbank with accession number MT773639.

We annotated the complete mitochondrial genome sequence of Nixi chicken by tRNAscan-SE 2.0 (Chan and Lowe 2019) and MITOS (Bernt et al. 2013) using the total length of 16,784 bp. The mitogenome has a typical vertebrate double-strand circular structure, containing one D-loop region, 2 ribosomal RNA genes (rRNAs), 13 protein-coding genes (PCGs) and 22 transfer RNA genes (tRNAs). All these genes have 10 overlaps in the length of 1–10 bp. Most genes (2 rRNAs, 12 PCGs and 14 tRNAs) are encoded on the heavy strand, while the rest genes (1 PCG and 8 tRNAs) are encoded on the light strand. Among 13 PCGs, *ND5* (1818 bp) is the longest while *ATP8* (165 bp) is the shortest PCG. The initiation codon of most PCGs is ATG except for *COX1* being GTG. There are four types of stop codons which are TAA, TAG, AGG and incomplete stop codon T, which is due to the 5′ terminal of the adjacent gene (Anderson et al. 1981). The 22 tRNA genes are distributed among rRNA and PCGs, ranging from 66 to 76 bp in length. The lengths of 12S rRNA and 16S rRNA genes are 977 bp and 1572 bp, respectively.

The phylogenetic position of Nixi chicken is revealed by constructing the neighbor-joining (NJ) phylogenetic tree together with other downloaded chicken mitogenomes from NCBI using MEGA 7.0 (Kumar et al. 2016) with 1000 bootstrap replicates. The NJ tree (Figure 1) indicates that Nixi chicken has the closest maternal relationship with Lverwu and Tibetan. However, Nixi chicken has the farthest genetic distance with Taoyuan. Our work provides a valuable source of data for the study of the evolution of the Gallus gallus mitochondrial genome and contributes to the Nixi chicken breeding improvement program.

**Figure 1.**
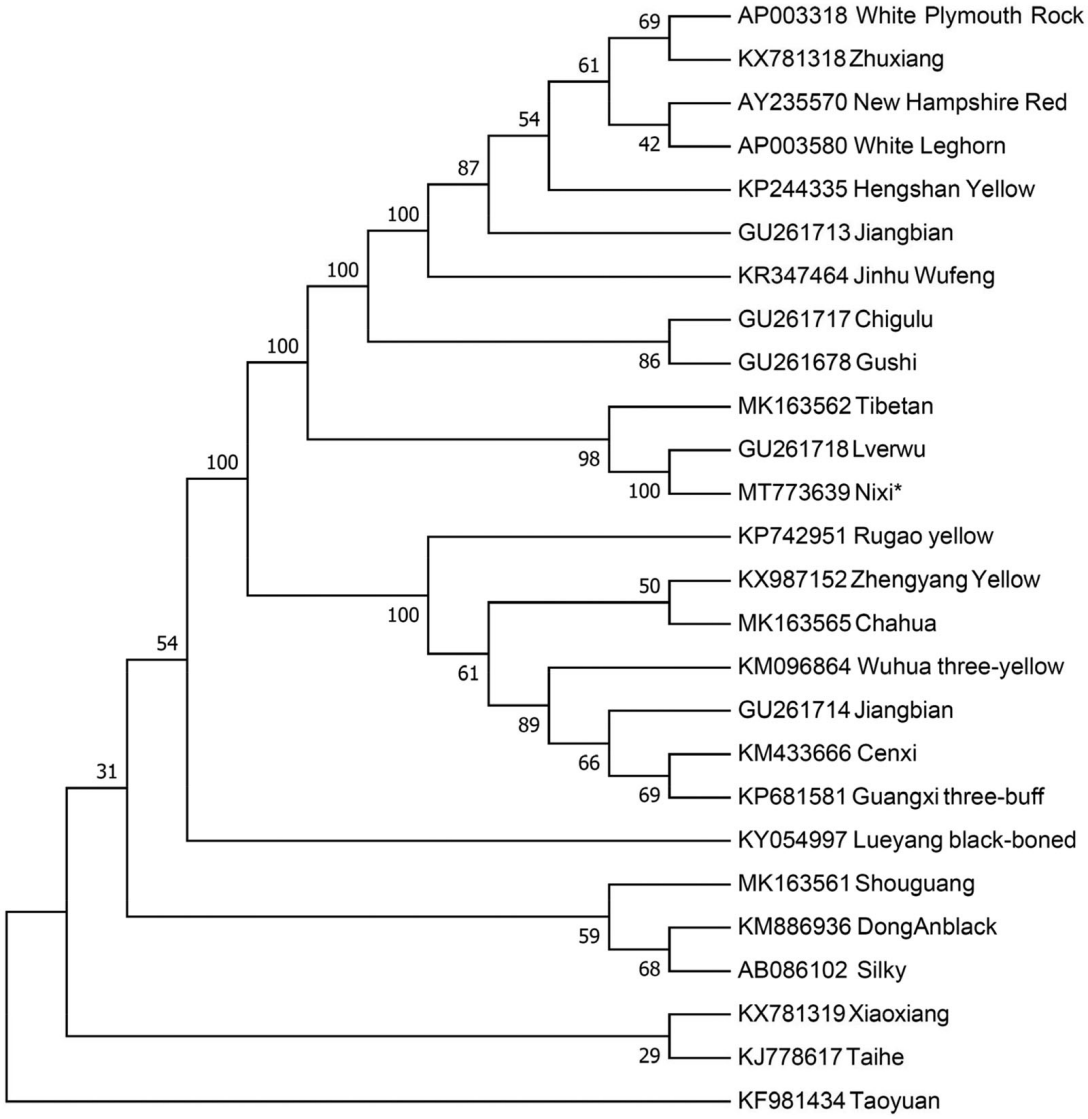
Neighbor-joining tree based on the complete mitochondrial DNA sequence of 26 chicken breeds. GenBank accession numbers are given before the species name.

## Data Availability

The sequence data that support the findings of this study are openly available in the NCBI Sequence Read Archive (SRA) at http://www.ncbi.nlm.nih.gov/sra/ with accession number SRR4302064. The complete mitochondrial genome of Nixi chicken (Gallus gallus) is openly available in GenBank at http://www.ncbi.nlm.nih.gov/genbank with accession number MT773639.
